# Reduction in the risk of contrast-induced nephropathy using enhanced external counter-pulsation in patients with chronic kidney disease

**DOI:** 10.1080/0886022X.2024.2396449

**Published:** 2024-09-25

**Authors:** Chun-Mei Zeng, Hong-Yu Pan, Yan-Mei Zhao, Zheng Ling, Ming Liu, Ying Feng

**Affiliations:** Department of Cardiology, Yulin First People’s Hospital (The Sixth Affiliated Hospital of Guangxi Medical University), Yulin, Guangxi, China

**Keywords:** Enhanced external counter-pulsation, chronic kidney disease, contrast-induced nephropathy, efficacy

## Abstract

**Objective:**

To evaluate the efficacy of EECP in the prevention of contrast-induced nephropathy (CIN) in patients with chronic kidney disease (CKD).

**Methods:**

A prospective trial was undertaken in the participants. A total of 280 patients with an estimated glomerular filtration rate (eGFR) of <60 ml/min/1.73 m^2^ who underwent percutaneous coronary artery procedures were enrolled and divided into two groups: the control group (*n* = 100) and the EECP group (*n* = 180). All patients received extracellular fluid volume expansion therapy with 0.9% normal saline, and patients in the EECP groups were also treated with EECP. The renal function indexes of the two groups were determined 48–72 h after coronary artery procedures.

**Results:**

In the EECP group, the BUN and serum creatinine (Scr) after coronary artery procedures were significantly lower than those before coronary artery procedures (BUN: 8.4 ± 3.5 *vs.* 6.6 ± 2.7 mmol/L, *p* < 0.001; Scr: 151.9 ± 44.7 *vs.* 144.5 ± 48.3 μmol/L, *p* < 0.001), while the eGFR was significantly increased (43.6 ± 11.4 *vs.* 47.1 ± 13.9 ml/min/1.73 m^2^, *p* < 0.001). The degree of Scr elevation was lower in the EECP group than in the control group (12.4 ± 15.0 *vs.* 20.9 ± 24.8 μmol/L, *p* = 0.026). Additionally, the EECP group had a lower incidence of post-procedures Scr elevation than the control group (36.5 *vs.* 48.0%, *p* = 0.042), a higher incidence of post-procedures eGFR elevation (62.2 *vs.* 48.0%, *p* = 0.021), and a lower risk of CIN (1.1 *vs.* 6.0%, *p* = 0.019).

**Conclusion:**

EECP therapy has a protective effect on renal function and can reduce the risk of CIN in patients with CKD.

## 1. Introduction

Contrast-induced nephropathy (CIN) is a kind of acute renal function injury caused by contrast agents. With the extensive development of percutaneous coronary intervention (PCI) in recent years, the number of patients with CIN has increased significantly, especially among patients with chronic kidney disease (CKD), which is one of the risk factors for CIN [1]. In patients with CKD, the risk of CIN is significantly increased even when contrast-enhanced CT examination is performed with a small volume of contrast agents [2], while the risk of CIN in patients undergoing PCI can reach more than 25% [3].

Extracellular fluid volume expansion therapy can expand blood volume, reduce the concentration and viscosity of the contrast agent, reduce the retention time of the contrast agent in the kidney, and accelerate the excretion of the contrast agent, thus reducing the incidence of CIN in the population from 3.3 to 1.4%. Hence, it is currently recognized as an effective method for CIN prevention [4].

Enhanced external counter-pulsation (EECP) is used in the treatment of ischemic diseases, such as angina pectoris, myocardial infarction, and cerebral infarction [5], by sequentially pressurizing the air sacs surrounding the calves, thighs, and hips through ECG triggering. In the present study, the efficacy of EECP therapy in the prevention of CIN in patients with CKD was evaluated by comparing the changes in renal function-related indicators in patients with CKD after coronary artery procedures.

## 2. Materials and methods

### 2.1. General data and methods

A prospective trial was undertaken in the participants. Patients with an estimated glomerular filtration rate (eGFR) of <60 mL/min/1.73 m^2^ who underwent coronary artery procedures (not on emergency settings) at our hospital between December 2020 and December 2021 were selected as the study subjects. All patients were measured for height and weight and examined for blood urea nitrogen (BUN), serum creatinine (Scr), and GFR within 72 h before coronary artery procedures. This study protocol complies with the ethical principles of the World Medical Association Declaration of Helsinki, approved by the ethics committee of our hospital (YLSY-IRB-KY-2021014). By reviewing the literature, the incidence of CIN in patients with eGFR < 30 mL/min/1.73 m^2^ was 38% [6], and the subjects enrolled in this study had eGFR < 60 mL/min/1.73 m^2^. We estimated that the incidence of CIN after coronary artery procedures in the control group would be about 20.0%. The pretest for this study showed that the incidence of CIN after coronary artery procedures in the EECP group was about 2.0%, with the sample size ratio of the control group and EECP group (1:2). By setting *α* = 0.05 (bilateral) and power = 0.85, the total sample size calculated according to the PASS 15.0 was 143 cases, including 48 cases in the control group and 98 cases in the EECP group. There were 293 participants. After 12 patients who failed to receive the reexamination of renal function indexes on time and one patient who underwent hemodialysis on the day after coronary artery procedures were excluded from the study, 100 patients in the control group and 180 patients in the EECP group were selected.

We recommended EECP treatment for all patients. However, some patients refused to receive EECP treatment due to treatment difficulties, economy, comfort, and other reasons, so randomization was not possible at that time. The patients were divided into two groups according to their wishes: the extracellular fluid volume expansion group (control group) and the extracellular fluid volume expansion + EECP group (EECP group). All patients received routine extracellular fluid volume expansion therapy with 0.9% normal saline [1.0–1.5 mL/(kg·h); 0.5 mL/(kg·h) in patients with a left ventricular ejection fraction of <35%] from 6–12 h before to 6–12 h after coronary artery procedures. Patients in the EECP group underwent EECP on the basis of extracellular fluid volume expansion; namely, EECP was performed 24 h before and after coronary artery procedures (once a day, 1 h each time, until 48–72 h after coronary artery procedures). The ratio of extracorporeal counterpulsation is set to 1:1, and the inflation pressure is adjusted between 0.020 and 0.035 MPa. The inflation and exhaust time is adjusted to make the ratio of diastolic pressure wave (D) to systolic pressure wave (S) as much as possible (D/S) >1.2, and the ratio of diastolic pressure area to systolic pressure area (DP/SP) is 1.5–2.0, depending on the patient’s tolerance.

Patients received intervention treatment after coronary angiography if they needed. All contrast agents used in this study were nonionic, including Iodixanol, Iohexol, and Ioversol. Patients in both groups were examined for Scr, BUN, eGFR, serum cystatin C at 48–72 h after coronary artery procedure. To investigate the effects of EECP on renal tubular function and inflammatory status, we simultaneously detected serum β 2 microglobulin, serum retinal binding protein, and hypersensitive C-reactive protein (Hs CRP); changes in renal function indexes and the risk of CIN were compared between the two groups.

Diagnostic criteria for CIN: An absolute Scr elevation of ≥0.5 mg/dL (44.2 µmol/L) or a 25% elevation relative to the baseline within 48–72 h after the use of an iodinated contrast agent.

Inclusion criteria: Patients aged ≥18 years; Patients with an eGFR of <60 mL/min/1.73 m^2^; Patients who underwent coronary artery procedures; patients with complete results of BUN, Scr, and eGFR within 72 h before coronary artery procedures; Patients who gave their informed consent.

Exclusion criteria: Patients who had used an iodinated contrast agent 30 days before enrollment; Patients with acute kidney injury due to other clear causes; patients who asked to withdraw from treatment; Patients who failed to receive the reexamination of renal function indexes on time after coronary artery procedures; Patients who underwent hemodialysis within 48 h after coronary artery procedures; Patients with uremia who received chronic hemodialysis.

### 2.2. Statistical methods

All the data collected in this study were analyzed using SPSS 22.0 software. Normally distributed measurement data were expressed as mean ± standard deviation (*SD*), while non-normally distributed measurement data were expressed as median (interquartile range), and the comparisons were examined by Student *t*-test and Mann–Whitney test (non-parametric distribution). The categorical data were expressed as *n* (%), and the differences between the two groups were examined by chi-square analysis or Fisher’s exact test. *p* < 0.05 was considered statistically significant.

## 3. Results

### 3.1. General data

There were no statistically significant differences in age, body mass index, complications, preoperative renal function indexes between the two groups; the data were comparable (Table 1).

**Table 1.** General data of patients.

General data	Control group (*n* = 100)	EECP group (*n* = 180)	*χ*^2^/*t*/*Z* value	*p*-Value
Gender [male (%)]	77 (77.0%)	141 (78.3%)	0.066	0.797
Age (years)	66.9 ± 9.4	67.9 ± 9.5	−0.783	0.434
BMI (kg/m^2^)	24.0 ± 4.0	24.0 ± 3.6	0.273	0.785
Smoking [*n* (%)]	33 (33.0%)	49 (27.2%)	1.036	0.309
Hypertension [*n* (%)]	79 (79.0%)	140 (77.8%)	0.056	0.812
Diabetes mellitus [*n* (%)]	29 (29.0%)	45 (25.0%)	0.529	0.467
LVEF < 35%	11 (11.0%)	10 (5.6%)	2.747	0.097
BUN (mmol/L)	8.1 ± 2.9	8.4 ± 3.5	−0.623	0.534
Scr (μmol/L)	147.3 ± 42.9	151.9 ± 44.7	−0.847	0.398
eGFR (ml/min/1.73 m^2^)	44.9 ± 10.7	43.6 ± 11.4	0.919	0.359
Statin [*n* (%)]	99 (99.0%)	175 (97.2%)	0.969	0.325
RAS inhibitor [*n* (%)]	89 (89.0%)	153 (85.0%)	0.877	0.349
PCI [*n* (%)]	68 (68.0%)	115 (63.9%)	0.480	0.488
Nonionic contrast agent			0.027	0.870
Iso-osmolar [*n* (%)]	41 (41.0%)	72 (40.0%)		
Low osmolar [*n* (%)]	59 (59.0%)	108 (60.0%)		
Total extracellular fluid volume expansion amount (ml/kg)^a^	15.4 (12.5, 16.7)	15.9 (13.8, 18.8)	−1.922	0.055
Duration of PCI (min)^a^	55.0 (30.0, 70.0)	50.0 (25.0, 70.0)	−1.246	0.213

EECP: enhanced external counter-pulsation; BMI: body mass index; LVEF: left ventricular ejection fraction; BUN: blood urea nitrogen; Scr: serum creatinine; eGFR: estimated glomerular filtration rate; PCI: percutaneous coronary intervention.

^a^Indicates M (Q_L_, Q_U_).

### 3.2. Comparison of post-procedures renal function indexes between the two groups

The levels of BUN and Hs-CRP were lower in the EECP group than in the control group (6.6 ± 2.7 *vs.* 7.5 ± 4.4 mmol/L, *t* = 2.111, *p* = 0.036 and 16.6 ± 26.6 *vs.* 31.0 ± 48.0 mg/L, *t* = 2.651, *p* = 0.009); however, there were no significant differences between the two groups in terms of Scr, eGFR, serum β2 microglobulin, cystatin, and serum retinol-binding protein (*p* > 0.05, Table 2).

**Table 2.** Comparison of post-procedures renal function indexes between the two groups.

Items	Control group (*n* = 100)	EECP group (*n* = 180)	*χ*^2^/*t* value	*p*-Value
BUN (mmol/L)	7.5 ± 4.4	6.6 ± 2.7	2.111	0.036
Scr (μmol/L)	149.5 ± 51.6	144.5 ± 48.3	0.800	0.425
eGFR (ml/min/1.73 m^2^)	45.3 ± 12.8	47.1 ± 13.9	−1.069	0.286
Serum β2-microglobulin (mg/L)	3.5 ± 1.4	3.4 ± 1.4	0.385	0.700
Cystatin-C (mg/L)	1.7 ± 0.6	1.6 ± 0.5	1.681	0.094
Serum retinol-binding protein (mg/L)	45.8 ± 14.4	47.5 ± 14.0	−0.931	0.353
Hs-CRP (mg/L)	31.0 ± 48.0	16.6 ± 26.6	2.651	0.009

EECP: enhanced external counter-pulsation; BUN: blood urea nitrogen; Scr: serum creatinine; eGFR: estimated glomerular filtration rate; Hs-CRP: hypersensitive C-reactive protein.

### 3.3. Comparison of renal function levels before and after coronary artery procedures

**Figure 1.**
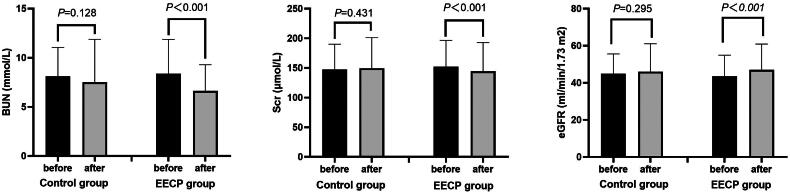
Comparison of renal function levels before and after coronary artery procedures.

There were no statistical differences in renal function indexes in the control group before and after coronary artery procedures (*p* > 0.05, Figure 1). The BUN and Scr decreased significantly (8.4 ± 3.5 *vs.* 6.6 ± 2.7 mmol/L, *t* = 8.426, *p* < 0.001 and 151.9 ± 44.7 *vs.* 144.5 ± 48.3 μmol/L, *t* = 4.356, *p* < 0.001), and the eGFR increased significantly (43.6 ± 11.4 *vs.* 47.1 ± 13.9 mL/min/1.73 m^2^, *t* = −5.288, *p* < 0.001) in the EECP group after coronary artery procedures (Figure 1).

### 3.4. Comparison of CIN risk between the two groups

In the EECP group, the proportion of post-procedures elevation of Scr was lower (35.6 *vs.* 48.0%, *χ*^2^ = 4.148, *p* = 0.042), and the proportion of post-procedures elevation of eGFR was higher (62.2 *vs.* 48.0%, *χ*^2^ = 5.310, *p* = 0.021) than in the control group, indicating a lower risk of CIN (1.1 *vs.* 6.0%, *χ*^2^ = 5.536, *p* = 0.019) in the EECP group (Table 3).

**Table 3.** Comparison of the incidence of contrast-induced nephropathy between the two groups.

Items	Control group (*n* = 100)	EECP group (*n* = 180)	*χ*^2^/*t* value	*p*-Value
Change of Scr			4.148	0.042
Increase	48 (48.0%)	64 (35.6%)		
Decrease	52 (52.0%)	116 (64.4%)		
ΔScr increase (μmol/L)	20.9 ± 24.8	12.4 ± 15.0	2.250	0.026
ΔScr increase %	13.8 ± 15.1	8.0 ± 6.8	2.478	0.016
CIN [*n* (%)]	6 (6.0%)	2 (1.1%)	5.536	0.019

EECP: enhanced external counter-pulsation; BUN: blood urea nitrogen; Scr: serum creatinine; eGFR: estimated glomerular filtration rate; CIN: contrast-induced nephropathy, diagnostic criteria: an absolute Scr elevation of ≥0.5 mg/dL (44.2 µmol/L) or a 25% elevation relative to the baseline within 48–72 h after the use of an iodinated contrast agent.

## 4. Discussion

The incidence of CIN is increasing with the rapid development of CT examination and interventional therapy. The incidence of CIN is relatively low (1–2%) in patients with normal renal function [3], but significantly higher in patients with CKD. Contrast agents constitute the main cause of iatrogenic kidney injury. Hemodialysis is required for ∼1% of patients with CIN during hospitalization, of whom ∼13% require lifetime hemodialysis, resulting in prolonged hospitalization, increased short-term and long-term mortality, and an accelerated progression of underlying CKD [7].

The osmotic effect of contrast agents will increase the transportation and reabsorption of Na^+^, further aggravating the burden of renal tubules. Oxidative stress caused by organ injury will activate inflammatory mediators [8,9], leading to degeneration and vacuolization of renal tubular epithelial cells, and protein deposition will block renal tubules, thereby aggravating renal damage.

With the lack of specific therapy for CIN, prevention is crucial. In early extracellular fluid volume expansion therapy, 0.45% normal saline was often used; in contrast, the effect of 0.9% normal saline is believed to have better results than 0.45% normal saline in current research. In addition, statins, acetylcysteine, and nicorandil have been shown to reduce the risk of contrast agent renal injury [10–12].

As a safe, non-invasive, and effective therapy, EECP is mainly used in the treatment and rehabilitation of angina pectoris, cerebral infarction, and chronic heart failure currently [13,14]; it can also improve erectile dysfunction in male patients [15]. Moreover, EECP has biological effects, such as regulating flow shear stress, improving vascular endothelial function, and inhibiting oxidative stress, thus improving myocardial perfusion [16] and protecting multiple organs.

An increasing number of scholars have been exploring the renal protective effect of EECP. According to the results of the study conducted by Werner, EECP could increase the eGFR by 24%, increase renal blood flow by 21–30%, and promote renal excretion function by reducing renal vascular resistance, renin activity, and endothelin [17]. Of course, Werner’s study population was cirrhosis patients with severe renal vasoconstriction, which was different from the population in this study. Whether EECP could achieve the same pathophysiological improvement needs further discussion.

The study conducted by Rua showed that EECP therapy could reduce serum cystatin by 0.06 mg/L (*p* < 0.001) and increase eGFR by 5.8 mL/min/1.73 m^2^; the effect was more significant in patients with renal insufficiency [18]. Affected by factors, such as diet and hemodynamic changes, some patients in both groups had improved renal function after PCI.

The results of this study support that EECP can protect the renal function of patients with CKD. After EECP treatment in patients, BUN and Scr decreased by 1.8 mmol/L and 7.4 µmol/L, respectively, and eGFR increased by 3.5 mL/min/1.73 m^2^; meanwhile, there were no significant changes in the renal function indexes of patients who received extracellular fluid volume expansion alone before and after coronary artery procedures.

Compared with patients who received extracellular fluid volume expansion therapy alone. The incidence of CIN was reduced from 6.0% in the patients who received extracellular fluid volume expansion therapy alone to 1.1% in those treated with extracellular fluid volume expansion + EECP, approaching the risk of CIN in people with normal renal function. Even when patients did experience deterioration in renal function, the deterioration was less severe than in patients treated with extracellular fluid volume expansion alone. A recent study showed that in patients with eGFR 60–89 mL/min/1.73 m^2^, EECP can improve the contrast agent clearance rate and reduce the risk of CI-AKI [19]. The eGFR of the patients enrolled in this study was <60 mL/min/1.73 m^2^, which could better reflect the role of EECP in high-risk groups of CIN (CKD patients). In addition, the Hs-CRP of patients after coronary artery procedures was significantly lower in the EECP group than in the control group; this suggests that EECP could inhibit oxidative stress by reducing inflammatory mediators, thereby reducing the risk of CIN. Unfortunately, we did not see significant differences in serum beta2-microglobulin and serum retinol binding protein between the two groups, and the absence of pre-procedure indicators could also affect the presentation of the results. The renal blood perfusion of patients was not investigated in this study and may be the direction of further exploration.

EECP can increase mean arterial pressure, reduce plasma renin concentration, decrease renal vascular resistance and endothelin-1 levels, increase atrial natriuretic peptide levels, improve urinary flow rate, increase renal perfusion, promote renal stress, and enhanced diuresis [17]. EECP alleviates ultrastructural changes, such as cell membrane atrophy, blistering, marginalization, degeneration, and nuclear fragmentation by regulating genes containing Baculoviral IAP repeat containing protein 2 (BIRC2) and apoptotic enzyme activating factor 1 (Apaf-1), significantly reducing the cell apoptosis index [20]. The fluid shear stress generated by EECP upregulates the expression and functional maintenance of multidrug and toxic compound efflux transporter (MATE) by activating the nuclear factor E2 related factor 2 (Nrf2) pathway, improving the cytotoxicity of contrast agents [21,22]. Meanwhile, the increase in NO induced by EECP and the improvement of endothelial function can reduce contrast agent induced oxidative stress. And diuretic improvement can increase the patient’s urine output, reduce the viscosity of the contrast agent, reduce the formation of microthrombus, and improve renal ischemia.

However, this study also has a few limitations. First, due to the fact that the patients were scheduled to be discharged 2–3 days after coronary artery procedures, and considering factors, such as distance from their place of residence, transportation, cost, and patient willingness, renal function data from a few weeks later were not available. From previous studies, EECP has been shown to increase renal blood flow perfusion, promote renal excretion, and alleviate inflammatory response [17,18]. Theoretically, it has a protective effect on renal function. Another study has also shown that short-term EECP treatment can reduce renal damage caused by contrast agent exposure after enhanced CT examination [19]. If the duration of EECP treatment is extended, such as six times a week, 1 h each time, for 6 weeks, for a total of 36 h, the author has not seen any relevant literature reports on the impact on renal function. Secondly, the amount of contrast agent administered was not accurately recorded but was indirectly estimated using the duration of coronary artery procedures. In addition, no urine data was collected, such as urinary microalbumin and N-acetyl-β-D-glucosaminidase (NAG) and urine beta 2 microglobulin are indicators used to evaluate early renal function damage. Thirdly, due to the limitation of sample size, this study did not conduct multivariate regression analysis to control confounding factors. Fourthly, the majority of patients did not undergo Hs CRP testing upon admission, so preoperative Hs CRP data cannot be provided. From previous studies, Hs CRP levels have been found to be associated with adverse ischemic events after PCI, with a higher risk of all-cause mortality and cardiac death associated with high Hs CRP levels [23]. Fifthly, the protective effect of EECP on the kidney may have been mediated through improved cardiac function rather than through direct effects on the kidney, which was not evaluated in this study. Considering that the basic principle of EECP reducing CIN is still unclear, and the existence of many confounding factors, such as hydration volumes and the number of patients with low EF, our next research directions include expanding the sample size to conduct randomized controlled studies and exploring the effects of EECP on renal hemodynamics from a physiological perspective. In the future, we will address these limitations to clarify the protective effect of EECP on the renal function.

## 5. Conclusion

This study found that after EECP treatment, BUN, serum creatinine decreased, eGFR increased, and the incidence rate of CIN decreased compared with the control group. Thus, we think that EECP can improve the renal function through reducing BUN and serum creatinine in CKD patients after coronary artery procedures, increasing the eGFR, and decreasing the risk of CIN, which may require more studies in the future to confirm.

## Data Availability

The datasets used and/or analyzed during the current study available from the corresponding author on reasonable request.
